# Supplementary material for: The Impact of Alcohol on Sleep Physiology: A Prospective Observational Study on Nocturnal Resting Heart Rate Using Smartwatch Technology
Source: Nutrients. 2025 Apr 26;17(9):1470. doi: 10.3390/nu17091470 (PMC12073130; doi:10.3390/nu17091470)
Supplement: Supplementary file 1 [file nutrients-17-01470-s001.zip › nutrients-3580537-supplementary.pdf]

Suppplementary Material

Supplementary Table S1. Subgroup analyses for average nocturnal HR. Total N=40. Data are presented as mean (SD); unit is bpm. Comparisons between pre-exposure and alcohol exposurewere performed using paired two-sided t-test. Statistically significant p values are highlighted in bold.

	Pre	Alcohol	Post	P value
Female (N=25)	65.7 (8.9)	69.3 (8.1)	66.2 (8.5)	**0.005**
Male (N=15)	60.0 (8.7)	62.1 (8.9)	62.7 (10.4)	0.074
BMI <25 kg/m^2 (N=24)	63.2 (9.6)	66.9 (9.1)	64.7 (10.1)	**0.006**
BMI ≥25 kg/m^2 (N=16)	64.1 (8.7)	66.2 (9.1)	65.2 (8.2)	0.056
Smokers (N=10)	65.1 (9.6)	70.6 (8.2)	64.4 (7.1)	0.054
Non-smokers (N=30)	63.0 (9.1)	65.3 (9.0)	65.0 (10.1)	**0.005**
Active (N=27)	63.5 (10.0)	65.4 (9.5)	64.4 (10.3)	**0.023**
Inactive (N=13)	63.7 (7.5)	69.2 (7.6)	65.8 (7.0)	**0.013**
Drinkers (N=20)	63.6 (9.6)	67.1 (9.6)	64.7 (9.3)	**0.023**
Abstainers (N=20)	63.5 (8.9)	66.1 (8.6)	65.0 (9.5)	**0.010**
Beer exposition (N=21)	62.6 (8.9)	65.3 (9.0)	64.2 (9.5)	**0.008**
Wine exposition (N=19)	64.6 (9.6)	68.1 (9.0)	65.6 (9.3)	**0.029**
